# Creating Unidirectionally Macrochanneled Zirconia Bone Scaffolds with Elongated Microporous Frameworks via Vat Photopolymerization Using Phase-Separable, Photocurable Vehicle

**DOI:** 10.3390/ma19173565

**Published:** 2026-08-22

**Authors:** Jae-Hyung Park, Jae-Min Jung, Se-Mi Lee, Dong-Yeon Bae, Jongee Park, Young-Hag Koh

**Affiliations:** 1 School of Biomedical Engineering, Korea University, Seoul 02841, Republic of Korea; jhbest210@korea.ac.kr (J.-H.P.); optima3000@hanmail.net (J.-M.J.); leesemi@korea.ac.kr (S.-M.L.); 2020250005@korea.ac.kr (D.-Y.B.); 2 Interdisciplinary Program in Precision Public Health, Korea University, Seoul 02841, Republic of Korea; 3 Department of Metallurgical and Materials Engineering, Atilim University, Ankara 06830, Türkiye; jongee.park@atilim.edu.tr

**Keywords:** vat photopolymerization, porous, zirconia, camphene, bone scaffolds

## Abstract

Unidirectionally macrochanneled tetragonal zirconia polycrystals (TZP) scaffolds, comprising elongated microporous frameworks, were fabricated via vat photopolymerization (VP) using a solution of 75 wt% camphene and 25 wt% 1,6-hexanediol diacrylate (HDDA) as a phase-separable, photopolymerizable vehicle. The camphene/HDDA solution underwent phase separation at 5 °C, accompanied by the recrystallization and dendritic growth of camphene, thereby generating three-dimensionally interconnected camphene crystal networks enclosed by HDDA. Following the photopolymerization of HDDA, the scaffolds were freeze-dried to remove the camphene crystals and subsequently heat-treated for debinding to eliminate organic phases, including photopolymerized HDDA, the dispersant, and the photoinitiator. The effect of sintering temperature on the geometry of the micropores and the densification of the TZP walls was examined. The optimum sintering condition (1500 °C for 3 h) enabled the creation of elongated micropores with a volume fraction of 61.17 ± 0.90 vol%, surrounded by highly densified TZP walls. The fabricated scaffolds exhibited well-defined macrochannels arranged in a hexagonal pattern, separated by microporous frameworks. Their overall porosity was as high as 74.65 ± 0.73 vol%, owing to the high framework microporosity. Despite their high porosity, the scaffolds achieved a compressive strength of 53.10 ± 8.19 MPa and a compressive modulus of 364.38 ± 70.90 MPa.

## 1. Introduction

Porous bioceramics have been widely employed as bone scaffolds to restore the function of damaged or diseased bones by promoting new tissue formation on their surfaces [1,2]. In particular, calcium phosphate (CaP) ceramics [3,4,5,6] and bioglasses [7,8] are bioactive materials that effectively stimulate the attachment, proliferation, and differentiation of bone-forming (osteogenic) cells, while undergoing controlled biodegradation during bone regeneration. These unique characteristics can allow progressive replacement by newly formed bone tissue [4,5,6,7,8], while scaffold porosity and pore architecture can further influence osteogenesis, cellular responses, and angiogenesis [9,10]. However, their relatively low mechanical strength limits their use in load-bearing bones, such as vertebrae and long bones, which should withstand substantial mechanical loads during bone regeneration [2,11,12]. On the other hand, 3 mol% yttria (Y_2_O_3_)-doped zirconia (ZrO_2_), known as TZP, can provide exceptionally high mechanical properties—compressive strength (~2000 MPa), flexural strength (900–1200 MPa), and fracture toughness (7–10 MPa·m^1/2^)—along with good biocompatibility [13,14,15,16]. Consequently, porous TZP scaffolds are promising candidates for load-bearing bone scaffold applications [17,18]. However, because they remain permanently in the body without degradation, it is desirable to minimize the amount of implanted material while still ensuring sufficiently high mechanical properties [13,14,17].

One of the most effective strategies for achieving high specific mechanical properties at given porosities is the construction of periodic pore networks in a highly reproducible manner using 3D printing and additive manufacturing (AM) [19,20,21,22]. In addition, pore architectures can be flexibly designed by considering the loading directions applied to bones. Isotropic porous architectures, such as cubic [23,24], diamond [25,26], truncated octahedral [23,24], woodpile lattices [27,28], and triply periodic minimal surfaces (TPMSs) [29,30,31], effectively resist multidirectional loads. In contrast, unidirectionally macrochanneled structures exhibit superior mechanical performance under compression parallel to the macrochannel direction [32,33], making them particularly advantageous for replacing bones subjected to compressive loads, such as in spinal cage applications [34,35]. Furthermore, the incorporation of micropores into ceramic frameworks comprising porous scaffolds can not only enhance specific mechanical properties but also facilitate the transport of body fluids, growth factors, oxygen, and nutrients, thereby promoting new bone tissue formation [36,37,38,39,40].

**Figure 1:**
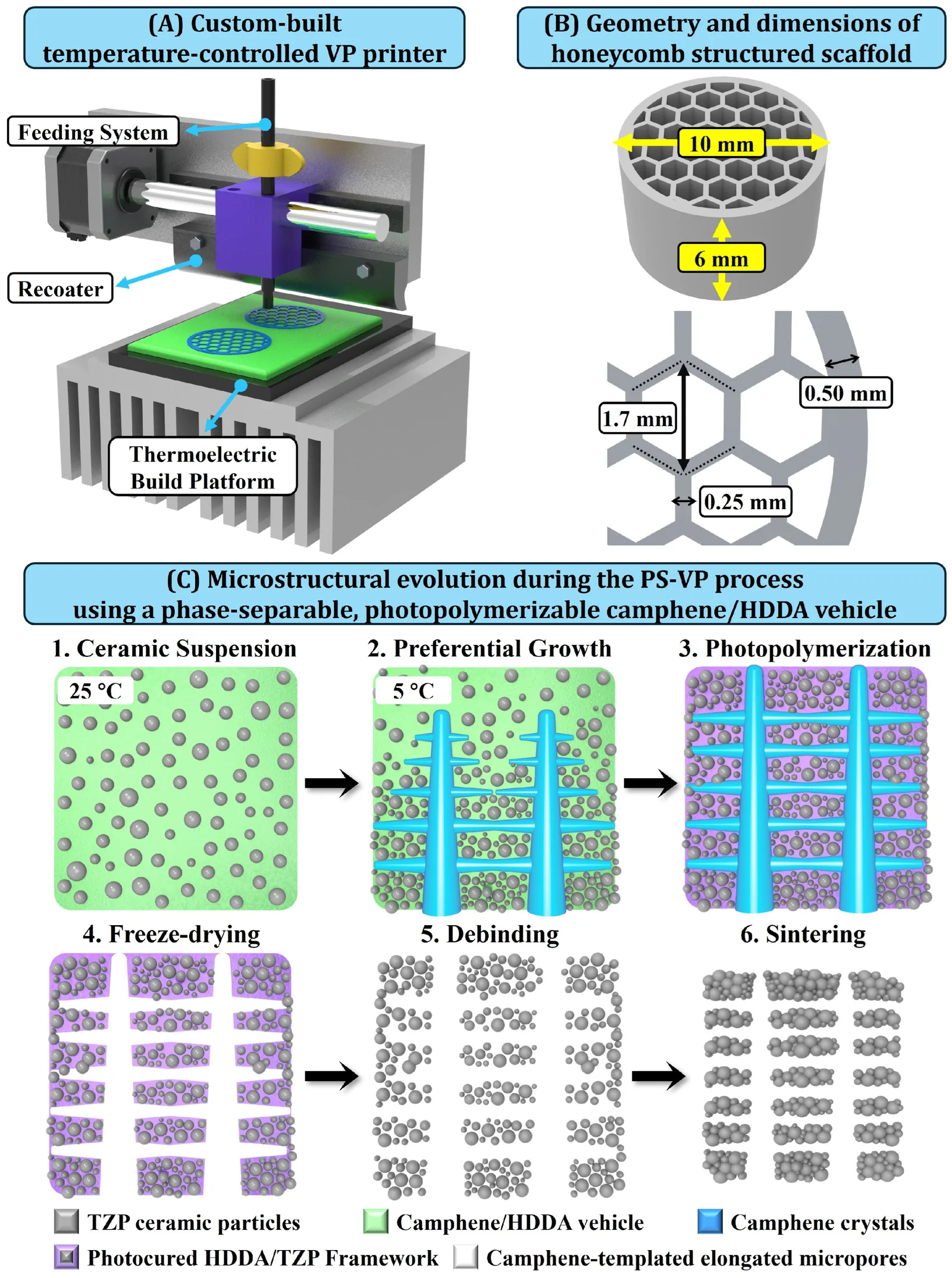
Schematic illustration of (**A**) a custom-built, temperature-controlled VP printer equipped; (**B**) the dimensions of the initial CAD model for fabricating unidirectionally macrochanneled scaffolds with macroporous frameworks; and (**C**) the sequential microstructural evolution throughout the PS-VP process and post-processes including freeze-drying, debinding, and sintering.

This study reports the fabrication of porous TZP bone scaffolds with high mechanical properties (compressive strength and modulus) at high porosity, composed of unidirectional macrochannels and microporous frameworks, via VP using a phase-separable, photopolymerizable vehicle. The fundamental concept of the VP process applied in this study, denoted as “phase separation-assisted VP (PS-VP)”, has been described in our previous report [36,41]. Figure 1A schematically illustrates the custom-built, temperature-controlled PS-VP printer equipped with a slurry-feeding system, a recoater, and a thermoelectric build platform. This printer was used to produce unidirectionally macrochanneled scaffolds consisting of periodic hexagonal macrochannels with microporous frameworks. The dimensions of the initial computer-aided design (CAD) model—including a macrochannel size of 1.7 mm, a framework width of 0.25 mm, and an outmost shell width of 0.5 mm—are detailed in Figure 1B. These dimensions were intentionally oversized in anticipation of sintering shrinkage to ensure a final pore size (~1 mm) suitable for bone ingrowth. The sequential microstructural evolution throughout the PS-VP process and post-processes (freeze-drying, debinding, and subsequent sintering) is depicted in Figure 1C. Thin layers of a homogeneous TZP suspension, prepared using a solution of 75 wt% camphene and 25 wt% HDDA monomer at room temperature, are spread onto the build platform by a recoater at 5 °C. This induces phase separation, during which camphene dissolved in HDDA recrystallizes and grows dendritically to form primary arms along the heat conduction direction, and secondary arms normal to the primary arms [41,42]. Simultaneously, liquid HDDA monomer containing TZP particles is displaced by the advancing camphene dendrites, resulting in a three-dimensionally interconnected network of camphene dendrites surrounded by an HDDA-TZP particle network. After phase separation, two-dimensional UV-illuminated images sliced from a predetermined CAD model were projected to the layers for photopolymerizing the HDDA–ceramic particle networks. The as-fabricated scaffolds were freeze-dried to remove camphene crystals via sublimation, resulting in a three-dimensionally interconnected pore network. The scaffolds were then subjected to heat-treatment for debinding to eliminate organic phases (photopolymerized HDDA, dispersant, and photoinitiator) through thermal decomposition, followed by densification of TZP particles at higher temperatures. To achieve desired microporous framework structures, different sintering temperatures (1350 °C, 1450 °C, and 1500 °C) were examined. The macroporous/microporous structures, and mechanical properties of unidirectionally macrochanneled TZP scaffolds with microporous frameworks were characterized to evaluate their potential as load-bearing bone scaffolds.

## 2. Materials and Methods

### 2.1. Compositions of TZP Suspension

The compositions and constituent volume fractions of TZP suspensions, composed of TZP powders, pore-forming agent, photocurable monomer, dispersant, and photoinitiator, are listed in Table 1. Unless otherwise specified, all reagents were used as received without further purification. Camphene (C_10_H_16_, Sigma-Aldrich, St. Louis, MO, USA) was employed as the pore-forming agent. 1,6-hexanediol diacrylate (HDDA, Tokyo Chemical Industry Co., Tokyo, Japan) was used as the photocurable monomer and solvent for camphene. DISPERBYK-180, an alkyloammonium salt of a block copolymer with acidic groups (BYK-Chemie Inc., Wesel, Germany), was used as the dispersant. Diphenyl(2,4,6-trimethylbenzoyl)phosphine oxide (TPO, Sigma-Aldrich, St. Louis, MO, USA) was used as the photoinitiator.

Meanwhile, commercially available yttria-stabilized zirconia powders (3YS-E, Tosoh Co., Tokyo, Japan) were used as the TZP source owing to their proper characteristics for the PS-VP process. Their morphology and particle size distribution were examined using field-emission scanning electron microscopy (FE-SEM; JSM-6701F, JEOL Techniques, Tokyo, Japan) and a laser diffraction particle size analyzer (LA-350, Horiba, Kyoto, Japan), respectively. Prior to FE-SEM observation, the powders were coated with gold using a magnetron sputter coater (108Auto, Cressington, Watford, UK) for 120 s at 20 mA under high vacuum to provide electrical conductivity and minimize charging during electron-beam irradiation. FE-SEM images were acquired at an acceleration voltage of 10 kV and a working distance of 8 mm.

### 2.2. Preparation of TZP Suspension

To obtain the highest camphene content required for achieving the highest porosity, the dissolution behavior of camphene in HDDA was investigated. Various camphene/HDDA mixtures with camphene weight ratios of 6:4, 7:3, 8:2, and 9:1 were examined. For each composition, predetermined amounts of solid camphene and liquid HDDA were weighed and heated at 50 °C until a visually homogeneous solution was obtained. After being kept at room temperature for more than 30 min, the mixtures were further cooled to initiate the formation of a small amount of camphene crystals. The partially crystallized mixtures were remixed using a vortex mixer (VM-96T, Jeio Tech Co., Ltd., Daejeon, Republic of Korea) and then placed again at room temperature. Digital photographs were taken to examine the optical appearances of the mixtures and to assess the dissolution state and mixing stability of camphene in HDDA.

TZP suspension was prepared by homogeneously mixing all constituents (TZP powder, camphene, HDDA, DISPERBYK-180, and TPO) at room temperature (cf., Table 1). To prepare TZP suspension, camphene was first dissolved in HDDA at room temperature to obtain a homogeneous camphene/HDDA solution. Subsequently, as-received TZP (3YS-E) powders were added to the camphene/HDDA solution together with DISPERBYK-180 as the dispersant. The mixture was homogenized using a planetary centrifugal mixer (Hantech Co., Ltd., Suwon-si, Gyeonggi-do, Republic of Korea) for 30 min at 1000 rpm to ensure uniform dispersion of the TZP particles. Prior to the DLP process, TPO was added as the photoinitiator, followed by additional mixing for 20 min at 1000 rpm. The prepared TZP suspension was used for PS-VP 3D printing without further treatment.

### 2.3. Characterization of TZP Suspensions

The rheological behavior of the TZP suspension was assessed by measuring its apparent viscosity as a function of shear rate using a cone/plate rheometer (DVNext, Brookfield Engineering Laboratories, Inc., Middleborough, MA, USA) at 25 °C. The shear rate was varied from 1 s^−1^ to 200 s^−1^ to evaluate the shear-dependent flow behavior of the suspension. Rheological measurements were repeated five times (*n* = 5).

To evaluate the photocuring behavior of the TZP suspension for the PS-VP process, suspension layers were placed on the temperature-controlled build platform and exposed to UV light using a digital light processing (DLP) engine (PRO4710, Wintech Digital Systems Technology Corp., San Jose, CA, USA) with a light intensity of 10.7 mW/cm^2^ at a peak wavelength of 405 nm. The resolution in the x–y plane was approximately 35.5 μm. The build platform was maintained at 5 °C to induce phase separation of the camphene/HDDA-based suspension prior to photopolymerization. To suppress unintended curing caused by reflected light, the platform surface was covered with black tape during UV exposure. For the photocuring depth measurement, the suspension layers were photocured for various exposure times (10–30 s), and the thicknesses of the photopolymerized layers were measured using a digital micrometer (293-821, Mitutoyo Corporation, Kawasaki-shi, Kanagawa, Japan) after rinsing with ethanol (94.5%, Daejung Chemicals & Metals Co., Ltd., Siheung-si, Gyeonggi-do, Republic of Korea) to remove uncured suspension. To evaluate the photocuring width and line broadening caused by UV scattering from the ceramic particles, long strips with designed widths ranging from 150 μm to 650 μm were photocured for 20 s. The widths of the photocured stripe patterns were then measured by FE-SEM. The photocuring depth and width measurements were repeated three times for each condition (*n* = 3).

### 2.4. Fabrication and Characterization of Microporous TZP Green Bodies

To examine the microporous structural evolution of TZP samples using the phase-separable, photopolymerizable camphene/HDDA solution, disks with a diameter of 8 mm and a thickness of 2 mm were fabricated using a temperature-controlled VP printer (cf., Figure 1). Specifically, 100-μm-thick TZP suspension layers, deposited on the build platform at 5 °C, were selectively photopolymerized for 20 s in a layer-by-layer fashion according to the sliced 2D images from a CAD model. The as-manufactured disks were detached from the build platform and rinsed with ethanol to remove uncured suspensions. Subsequently, the samples were freeze-dried for 24 h using a freeze dryer (ilShinBioBase Co., Ltd., Dongducheon-si, Gyeonggi-do, Republic of Korea) to remove the camphene dendrites. The overall morphology and microporous structure of the freeze-dried samples were examined by optical microscopy (OM) and FE-SEM, respectively.

### 2.5. Debinding and Sintering Processes

**Figure 2:**
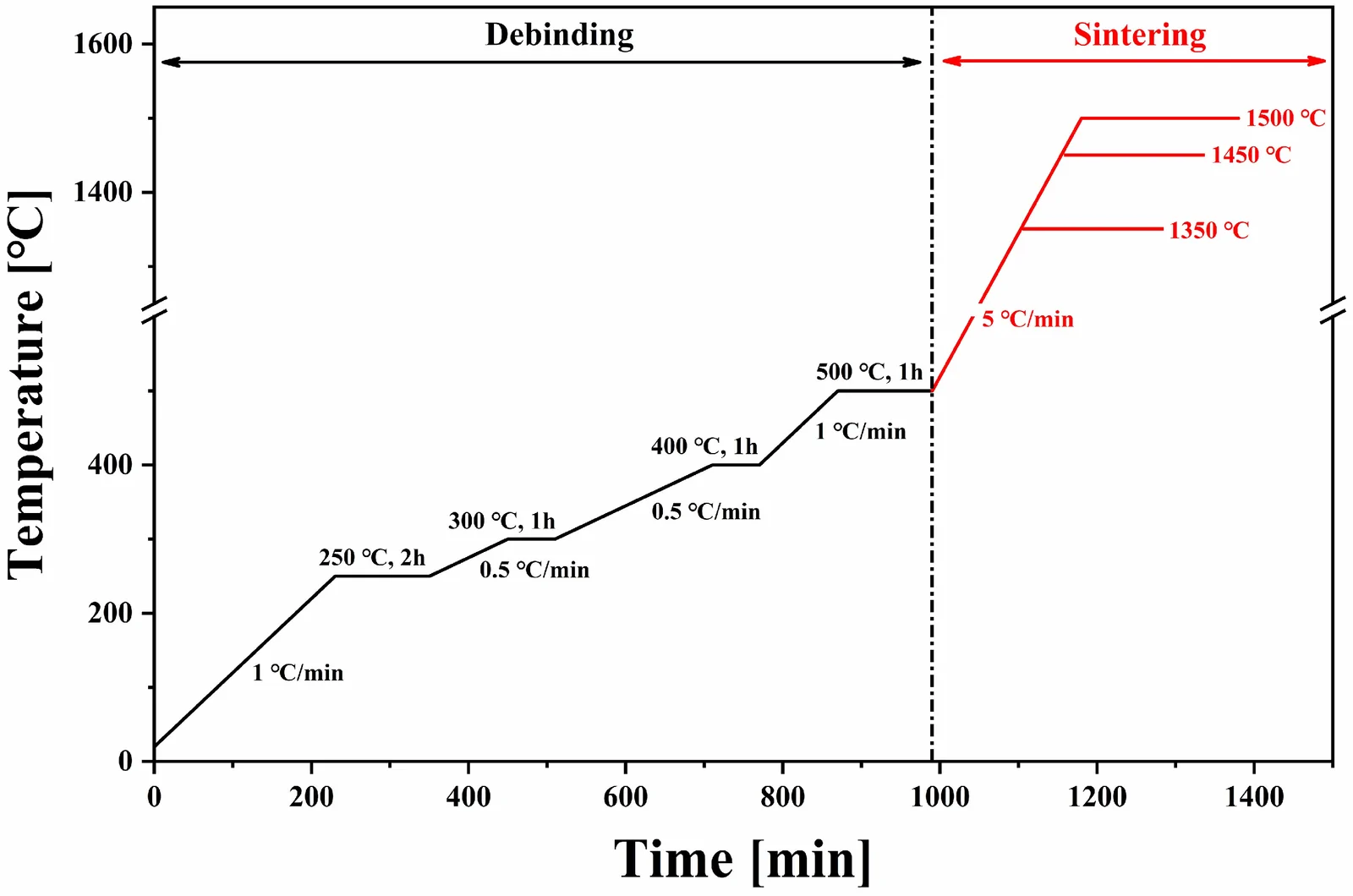
Heat-treatment schedule for the debinding and sintering of freeze-dried TZP samples, showing a multi-step debinding process up to 500 °C and subsequent sintering at various temperatures (1350 °C, 1450 °C, and 1500 °C).

The freeze-dried samples were heat-treated using a furnace (Ajeon Heating Industrial Co., Ltd., Namyangju-si, Gyeonggi-do, Republic of Korea) for debinding to remove organic phases (i.e., photopolymerized HDDA, dispersant, and photoinitiator), followed by densification of TZP particles via high-temperature sintering. The detailed heat-treatment schedule is illustrated in Figure 2. For debinding, a multi-step heat-treatment schedule was employed based on our previous study using a camphene/HDDA-based photocurable ceramic system [41], with slow heating rates particularly in the temperature range of approximately 250–500 °C to avoid crack formation associated with the extensive thermal decomposition of photopolymerized HDDA. Furthermore, compared to our previous study using alumina suspensions, slightly slower heating rates were employed at low temperatures (200 °C and 300 °C) particularly to remove the dispersant along with the photoinitiator, since a higher dispersant content was utilized in the TZP suspensions due to the higher density of TZP. Following the debinding stage, various sintering temperatures (1350 °C, 1450 °C, and 1500 °C) with a dwelling time of 3 h were applied to examine their effect on the pore structure of the microporous samples and the densification of the TZP walls.

### 2.6. Characterization of Microporous Disks

The microporous structures were characterized using FE-SEM. In addition, the grain size comprising the TZP walls was measured by the linear intercept method [43]. The crystalline phases coexisting in the 3YS-E particles were characterized by X-ray diffraction (XRD; D8 Advance, Bruker, Billerica, MA, USA) equipped with a Cu-Kα radiation source (λ = 1.5418 Å). XRD patterns were recorded over a 2θ range of 10–80° at a scan speed of 3° min^−1^ under operating parameters of 40 kV and 40 mA. The obtained diffraction patterns were indexed with reference to the standard patterns for tetragonal zirconia (t-ZrO_2_, JCPDS No. 01-079-1767) and monoclinic (m-ZrO_2_, JCPDS No. 01-081-1314) zirconia phases. To determine the weight fractions of the coexisting phases quantitatively, Rietveld refinement was conducted using XRD analysis software (Diffrac. Topas, Version 5.0, Bruker, Billerica, MA, USA).

The sintering shrinkage in the x- and z-directions were calculated based on the dimensional changes of the samples before and after sintering, as follows:

(1) $$Linear\;Shrinkage\;\left[\%\right]=100\;\cdot \left(\frac{Initial\;Dimension-Sintered\;Dimension}{Initial\;Dimension}\right)$$

The microporosity of the sintered samples was quantitatively evaluated from their relative densities. The mass of each specimen (m) was measured using an analytical balance (Pioneer, OHAUS Corporation, Parsippany, NJ, USA), and the geometric volume (V) was calculated from the measured diameter and height of the cylindrical samples. The bulk density $\left(\rho_{B}\right)$ was obtained as follows:

(2) $$\rho_{B}\;\left[g/{cm}^{3}\right]=m/V$$

The relative density $\left(\rho_{Rel}\right)$ and microporosity $\left(P_{Mi}\right)$ were calculated according to the following equations:

(3) $$\rho_{Rel}\;\left[\%\right]=100\cdot (\rho_{B}/\rho_{T})$$

(4) $$P_{Mi}\;\left[\%\right]=100-\rho_{Rel}$$

where $\rho_{T}$ is the theoretical density of TZP ceramic (6.05 g/cm^3^, according to manufacturer’s specifications). For each condition, three specimens were examined, and the results calculated using the above equations are presented as the mean and standard deviation (*n* = 3).

### 2.7. Fabrication and Characterization of Unidirectionally Macrochanneled Scaffolds with Elongated Microporous Frameworks

Unidirectionally macrochanneled TZP scaffolds, consisting of elongated microporous frameworks, were fabricated using the PS-VP process. The geometry of the initially designed scaffold is illustrated in Figure 1. The unit cell, arranged in a hexagonal pattern, had a size of 1.7 mm with a framework width of 0.5 mm. The diameter and height of the scaffold were 10 mm and 6 mm, respectively. The fabrication process was carried out using the same processing parameters as those used for the microporous disks: a layer thickness of 100 μm, a UV illumination time of 20 s at 5 °C for the PS-VP process, rinsing, freeze-drying, and post heat-treatment (debinding). However, sintering was conducted at 1500 °C for 3 h to achieve dense TZP walls surrounding the micropores within the TZP frameworks.

The overall structure and macrochanneled features were examined by optical microscopy. The total porosity $\left(P_{T}\right)$ of the scaffolds, including the macroporosity and microporosity, was calculated by measuring their mass and volume in a manner analogous to Equations (1)–(3). To calculate the macroporosity $\left(P_{Ma}\right)$, top-view images of the samples were captured and then processed using ImageJ software (Version 1.54g, National Institutes of Health, Bethesda, MD, USA). Since the macrochannels were unidirectionally aligned and penetrated throughout the entire scaffold height, the area fraction of the segmented macrochannels in the top-view image was regarded as the macroporosity, according to the following equation:

(5) $$P_{Ma}\;\left[\%\right]=100\;\cdot \left(\frac{A_{Ma}}{\;A_{T}}\right)\;$$

where $A_{Ma}$ is the area of the macrochannels in the top-view OM image, and $A_{T}$ is the cross-sectional area of the scaffold surface. A total of five scaffold specimens were tested, and the results are expressed as the mean and standard deviation (*n* = 5). The macroporous and microporous structures were examined by FE-SEM.

Compressive strength testing was conducted to evaluate the mechanical properties of unidirectionally macrochanneled TZP scaffolds with elongated microporous frameworks. The scaffolds were compressed using a universal testing machine (UTM; ST-1000, Salt Co., Ltd., Incheon, Republic of Korea) at a crosshead speed of 1 mm/min. The compressive load was applied parallel to the direction of the unidirectional macrochannels. The compressive strength and elastic modulus were calculated from stress–strain curves, and the fracture behavior after compression was examined by optical microscopy. A total of five scaffold specimens were tested, and the results are expressed as the mean and standard deviation (*n* = 5).

### 2.8. Statistical Analysis

Prior to parametric statistical analysis, the normality of the data distribution was verified using the Shapiro–Wilk test, and the homogeneity of variance was assessed using Levene’s test. All quantitative data are expressed as the mean and standard deviation. Statistical comparisons among groups were conducted using one-way analysis of variance (ANOVA) followed by Tukey’s post hoc test using RStudio (RStudio, Version 2026.01.2, Posit Software, PBC, Boston, MA, USA). Differences were considered statistically significant at *p* < 0.05 and are denoted by different superscript letters in the corresponding figures.

## 3. Results and Discussion

### 3.1. Characterization of TZP Powders

**Figure 3:**
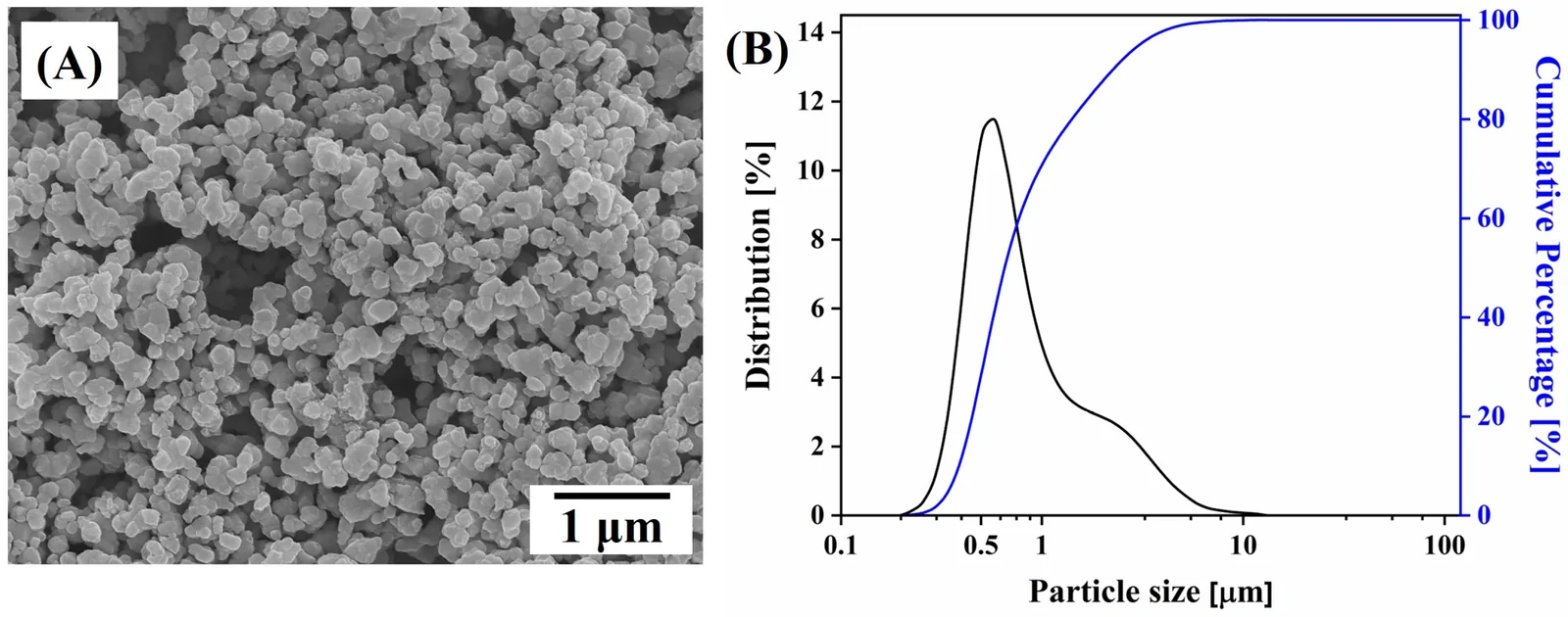
(**A**) FE-SEM image and (**B**) particle size distribution of the as-received 3YS-E powder, where the black and blue lines represent the particle size distribution and cumulative percentage, respectively.

To fully exploit PS-VP for fabricating unidirectionally macrochanneled scaffolds composed of the desired microporous frameworks, TZP particles should be sufficiently small to enable the recrystallization and dendritic growth of camphene during the phase separation of the camphene/HDDA solution, thereby generating elongated pores for high mechanical properties. To this end, commercially available 3YS-E powder was used as received for the preparation of TZP suspension in this study. As shown in Figure 3A, the as-received 3YS-E powder consisted of fine submicron-sized particles with partially agglomerated morphology. The particle size distribution was further examined by laser diffraction analysis, as shown in Figure 3B. The powder exhibited a main particle size distribution in the submicron range, with a broad distribution extending to several micrometers, which is likely associated with partial agglomeration of the fine TZP particles. These fine particles are effectively redistributed by the growing camphene dendrites during phase separation [36,41,42], enabling the formation of microporous TZP frameworks after photopolymerization and camphene removal.

### 3.2. Dissolution Behavior of Camphene in HDDA Monomer

**Figure 4:**
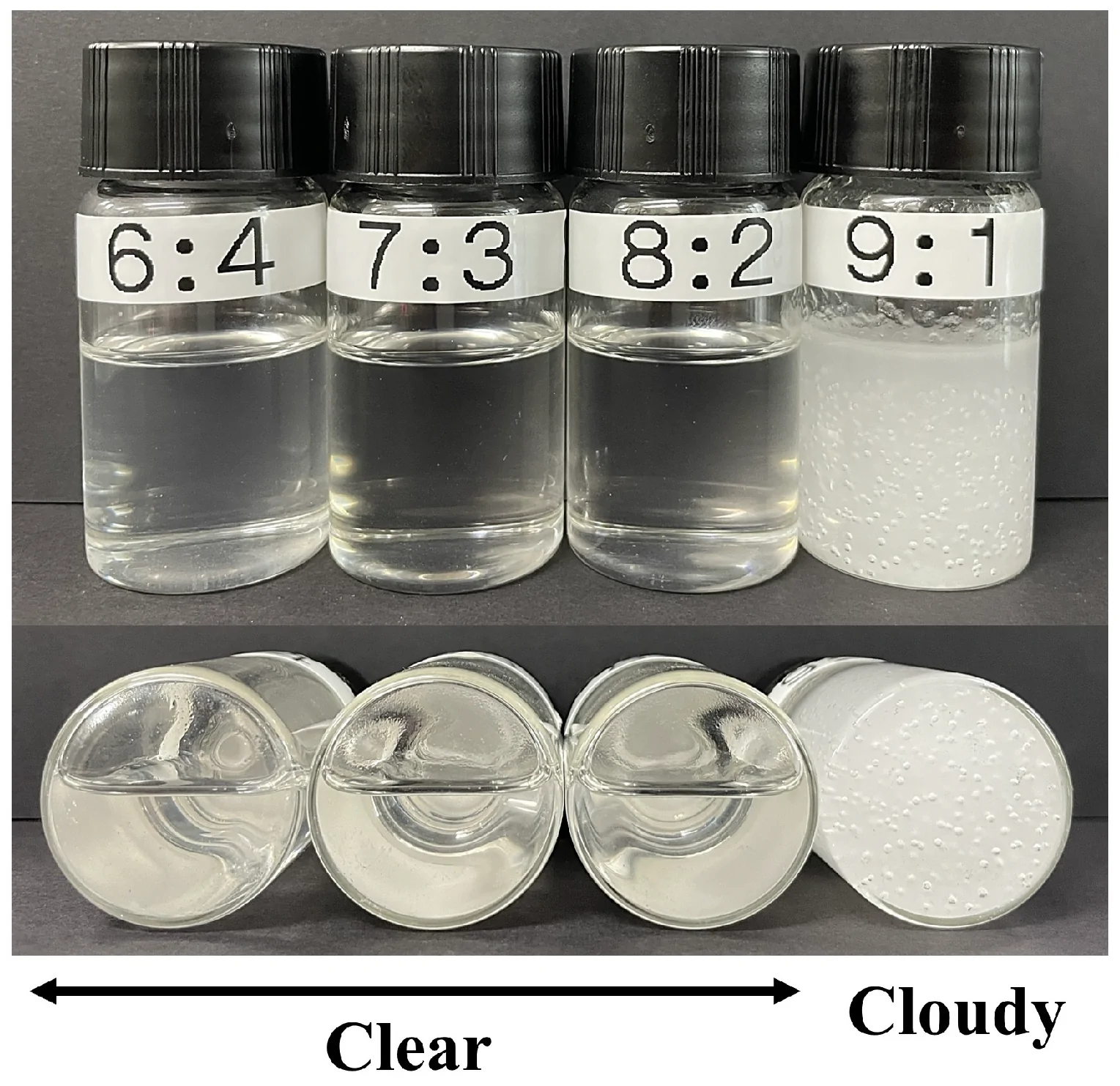
Optical images of camphene/HDDA mixtures with various camphene contents at room temperature. The numbers on the vials indicate camphene:HDDA weight ratios (6:4, 7:3, 8:2, and 9:1).

The dissolution behavior of camphene in HDDA was examined by preparing camphene/HDDA mixtures with various weight ratios at room temperature. As shown in Figure 4, the mixtures with camphene/HDDA ratios of 6:4, 7:3, and 8:2 remained transparent, indicating complete dissolution of camphene in HDDA without visible phase separation or precipitation. This result demonstrates that HDDA can effectively serve as both a photocurable monomer and a solvent for camphene in a phase-separable vehicle system [41]. In contrast, the 9:1 mixture became opaque, suggesting that the camphene content exceeded its solubility limit in HDDA at room temperature. Thus, the solubility limit of camphene in HDDA was considered to lie between 80 and 90 wt% camphene. Based on these results, a camphene/HDDA weight ratio of 7.5:2.5 was selected for the preparation of the TZP suspension in this study, as this composition maintained a homogeneous solution at room temperature while enabling camphene crystallization and dendritic growth upon cooling to 5 °C. This composition was therefore suitable for fabricating TZP scaffolds with interconnected microporous frameworks through the PS-VP process.

### 3.3. Rheological Behavior of TZP Suspension

**Figure 5:**
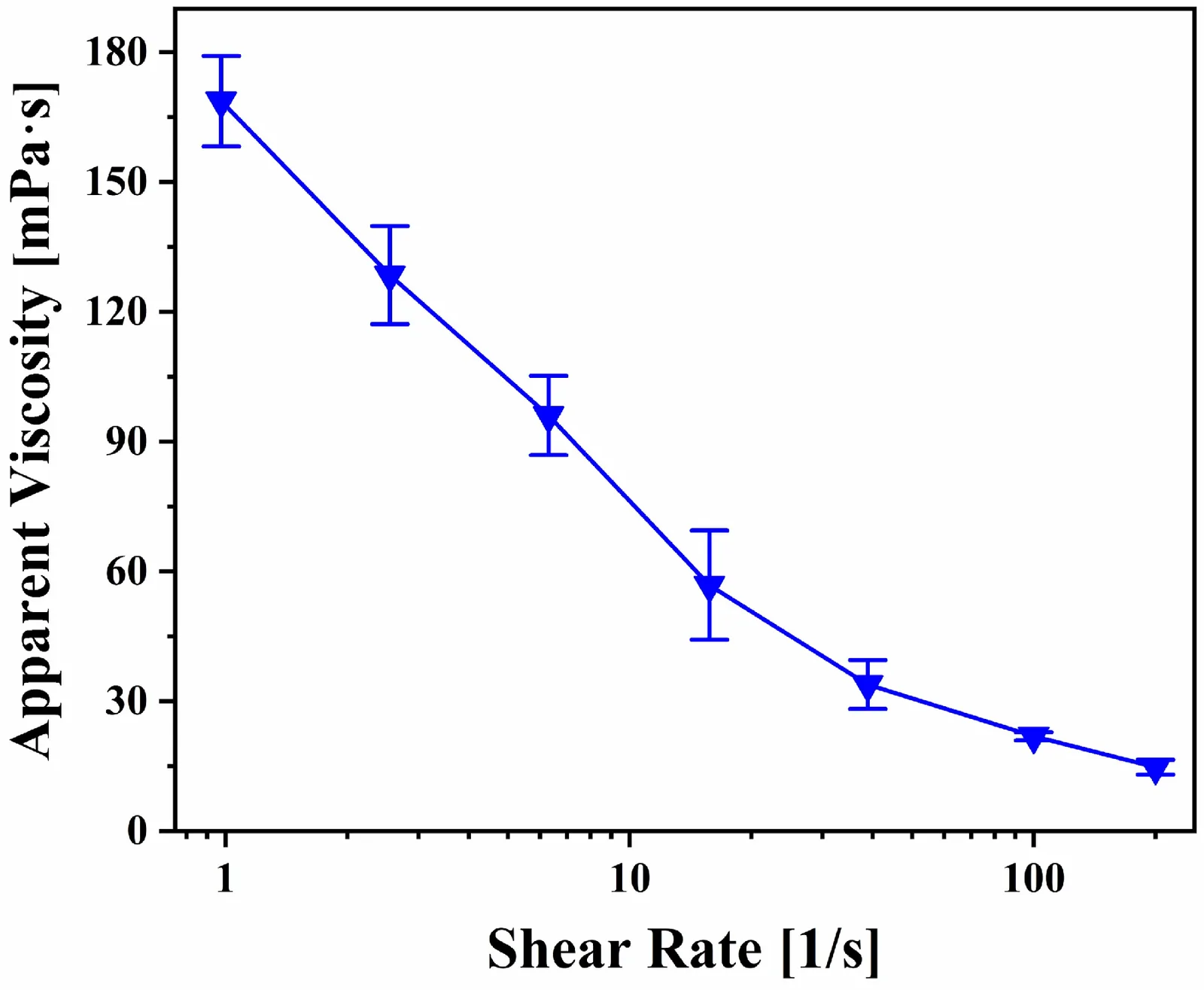
Apparent viscosity of the TZP suspension measured over a shear rate range of 1–200 s^−1^.

The rheological behavior of the TZP suspension is a critical factor in fabricating unidirectionally macrochanneled TZP scaffolds with elongated microporous frameworks via PS-VP, as it directly affects uniform layer coating and the removal of uncured suspension from the designed microchannel regions [44]. The apparent viscosity of the TZP suspension as a function of shear rate measured using a cone-plate viscometer is shown in Figure 5. The suspension exhibited typical shear-thinning behavior, where the apparent viscosity decreased with an increasing shear rate. The low viscosity at high shear rates (e.g., 21.9 mPa·s at 200 s^−1^) allowed the suspension to flow readily during recoating and to be easily removed from the uncured macrochannel regions after photopolymerization. Meanwhile, the coated layer after spreading maintained its geometry owing to the high viscosity at low shear rates (e.g., 168.7 mPa·s at 1 s^−1^). Furthermore, the camphene/HDDA-based suspension transformed into a semi-solid, gel-like state upon phase separation at 5 °C, which further supports thin-layer retention during the PS-VP process [36]. This finding indicates that the TZP suspension with desired rheological characteristics enables stable layer formation, effective macrochannel fabrication, and the subsequent development of elongated microporous frameworks.

### 3.4. Photopolymerization Behavior of TZP Suspension

**Figure 6:**
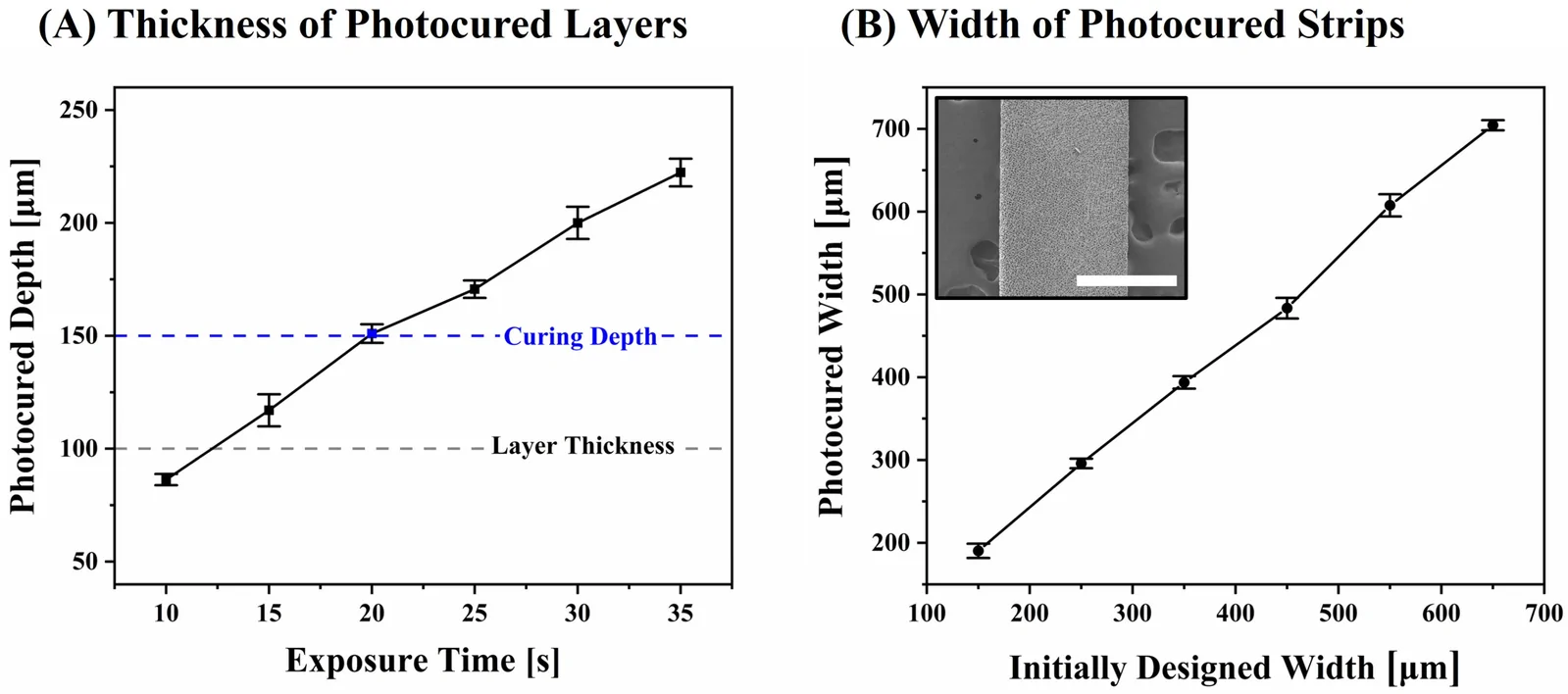
Photopolymerization behavior of the phase-separated TZP suspension at 5 °C: (**A**) photocured depth of TZP layers as a function of exposure time and (**B**) photocured width of strips as a function of the initially designed width after a 20 s exposure. The inset in Figure 6B shows a representative FE-SEM image of the manufactured strip (scale bar = 500 μm).

To determine the optimal photocuring time for complete photopolymerization of the TZP suspension layers and sufficient bonding between adjacent layers, the photocuring depth was measured as a function of UV exposure time. For this purpose, TZP suspension layers were photocured for various exposure times at 5 °C using a DLP engine, and the thicknesses of the photocured layers were measured after removing the uncured suspension. As shown in Figure 6A, the photocured depth increased from approximately 88 μm to 230 μm as the exposure time increased from 10 to 35 s. This result indicates that UV light can penetrate camphene crystals without severe attenuation and effectively photopolymerize HDDA monomer enclosing TZP particles. Based on these observations, a UV illumination time of 20 s, which can offer a cure depth of ~150 μm, was selected to ensure complete photopolymerization of the 100-μm-thick TZP suspension layers during the layer-by-layer PS-VP process. To evaluate line broadening caused by UV scattering from ceramic particles, long strips with various initially designed widths, ranging from 150 to 650 μm, were photocured for 20 s, and their final widths were measured by FE-SEM after freeze-drying (inset in Figure 6B). As shown in Figure 6B, the photocured widths were larger than the initially designed values over the entire width range, indicating the occurrence of line broadening during photopolymerization [45,46]. In addition, the measured photocured width increased almost linearly with increasing initially designed width. However, it should be noted that the final porous architecture of unidirectionally macrochanneled TZP scaffolds, including the unit cell size, macrochannel width, and framework width, can be controlled by carefully designing the initial CAD geometry in consideration of the line broadening effect during photopolymerization.

### 3.5. Microporous Structures of TZP Green Samples

To examine the microporous structural evolution of the TZP samples, disks with a diameter of 8 mm and a thickness of 2 mm were fabricated using the PS-VP process. Subsequently, the as-fabricated samples were freeze-dried for 24 h to remove camphene crystals. The weight loss was 43.61 ± 0.90 wt%, which was slightly lower than the initial camphene content (46 wt%) in the TZP suspension. This discrepancy is presumably due to the evaporation of camphene during the TZP suspension preparation and sublimation during the PS-VP process. However, the remaining camphene could be readily removed via sublimation in the solid state [43,47], enabling the maintenance of the 3D networks of HDDA enclosing the TZP particles.

**Figure 7:**
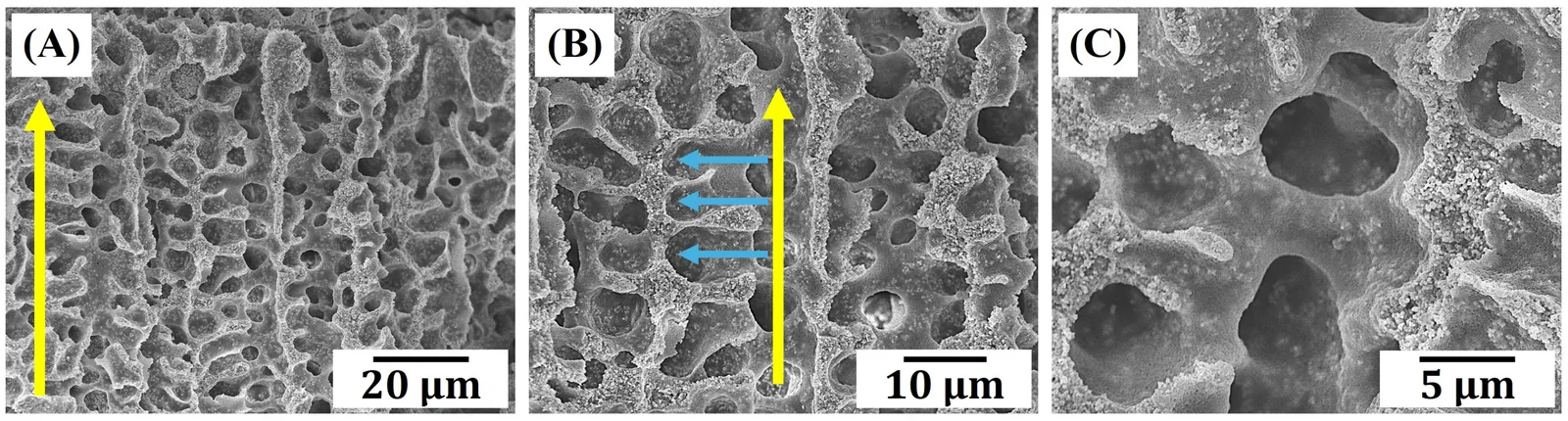
FE-SEM images of freeze-dried TZP samples, showing (**A**) the elongated pore architecture formed along the layer-by-layer PS-VP direction, marked by a yellow arrow, (**B**) pores oriented perpendicular to the elongated pores, marked by sky-blue arrows, and (**C**) the microstructure of HDDA/TZP particle walls.

Figure 7A–C present representative FE-SEM images of the porous structures and microstructure of the freeze-dried TZP samples. Highly elongated pores, marked by the yellow arrow in Figure 7A, were well formed along the layer-by-layer PS-VP direction. This finding indicates that the primary camphene crystals preferentially grew along the heat conduction direction, extending from the cooled build platform at 5 °C to the top surface of the TZP suspension. In addition, secondary arms were also developed perpendicular to the primary camphene crystals. Consequently, two types of elongated pore networks, marked by the yellow and sky-blue arrows in Figure 7B, were generated with excellent interconnection between the pores. The HDDA/TZP particle walls exhibited a smooth surface morphology and a relatively dense microstructure without any noticeable large voids or cracks (Figure 7C). Furthermore, the TZP particles were uniformly distributed within the photopolymerized HDDA frameworks. This microstructure suggests that the fine TZP particles were effectively redistributed by the growing camphene crystals, with assistance from the flowable liquid HDDA [41]. As a result, relatively dense HDDA/TZP particle frameworks could be formed after phase separation and photopolymerization, thereby enabling the subsequent densification of the TZP particles during high-temperature sintering and contributing to improved mechanical properties.

### 3.6. Effect of Sintering Temperature on Microporous Structure and Densification of TZP Walls

**Figure 8:**
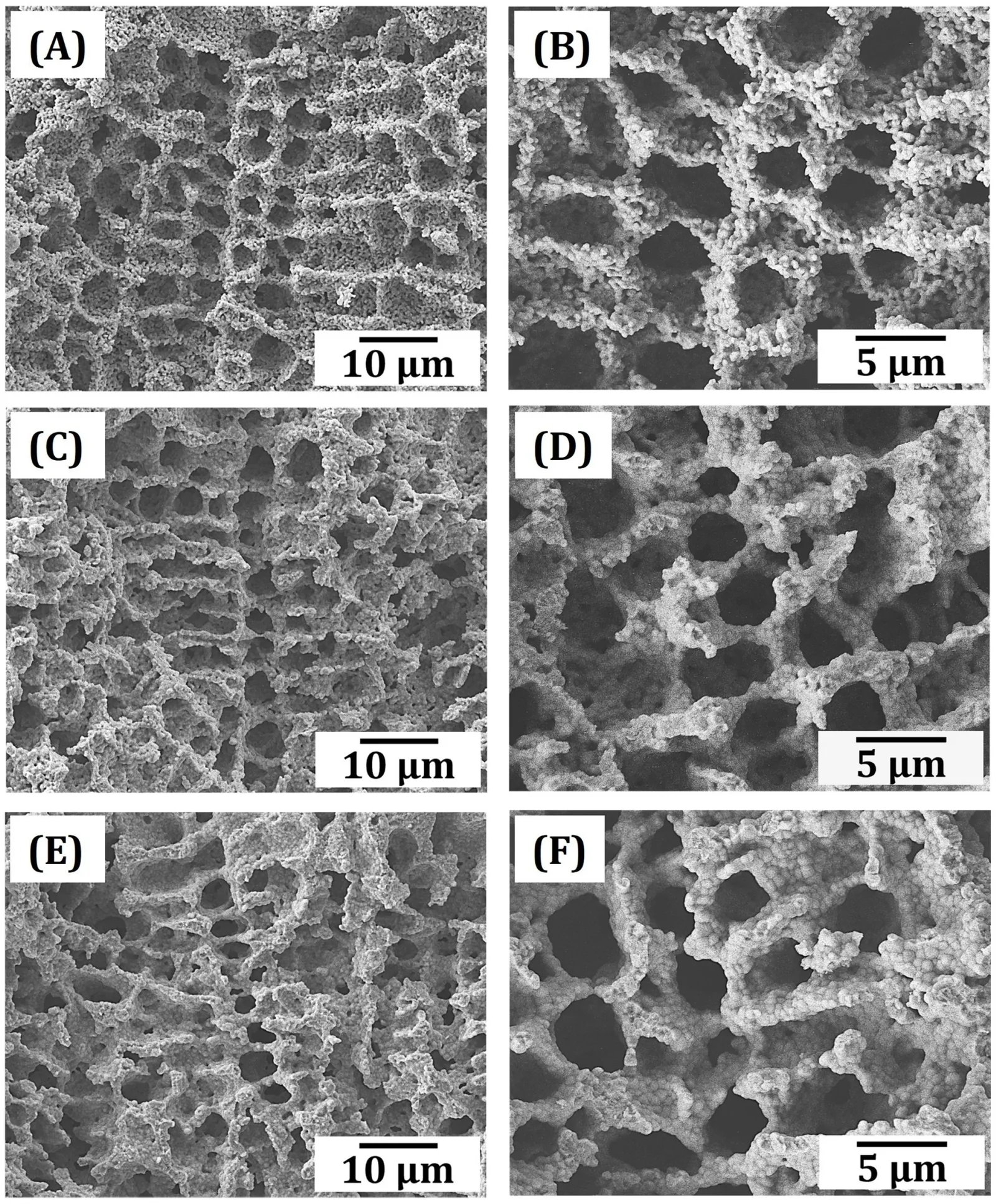
Representative FE-SEM images of TZP samples sintered for 3 h at various temperatures—1350 °C (**A**,**B**), 1450 °C (**C**,**D**), and 1500 °C (**E**,**F**)—showing their pore structures. The FE-SEM images were captured parallel to the layer-by-layer PS-VP direction.

The mechanical properties and performance of unidirectionally macrochanneled TZP scaffolds, consisting of microporous frameworks, are significantly influenced not only by the fractions of macrochannels and micropores, but also by the morphology of micropores and the densification of TZP walls. Optimizing the sintering temperature is therefore crucial to achieve highly densified TZP walls, while maintaining the elongated pores formed within the green bodies [48,49]. Prior to high-temperature sintering, the freeze-dried TZP samples were subjected to the multi-step debinding process (cf., Figure 2) to remove the organic phases, including the photopolymerized HDDA, dispersant, and photoinitiator. In this process, slow heating rates were applied particularly in the temperature range of approximately 250–500 °C, where extensive thermal decomposition of the organic components occurs, to avoid crack formation during debinding. After debinding, the microporous TZP samples were sintered at various temperatures (1350 °C, 1450 °C, and 1500 °C for 3 h) and their microporous structures as well as the densification of TZP walls were characterized. Representative FE-SEM images are presented in Figure 8A–F. All samples showed relatively long and short elongated pores parallel and perpendicular to the layer-by-layer PS-VP direction (Figure 8A,C,E). These observations indicate that the micropores in the green bodies, formed as replicas of primary and secondary camphene dendrite arms, were well preserved without any noticeable defects, such as cracks and voids, particularly owing to the optimized multi-step debinding process. However, the degree of densification of the TZP walls increased remarkably with increasing sintering temperatures, as shown in Figure 8B,D,F. When sintered at the lowest temperature of 1350 °C (Figure 8B), the TZP walls exhibited numerous small pores due to insufficient particle densification. These residual pores decreased significantly as the sintering temperature increased to 1450 °C (Figure 8D), although some pores still remained within the walls. In contrast, sintering at the highest temperature of 1500 °C (Figure 8F) yielded highly densified TZP walls with substantially reduced residual porosity. These results indicate that the sintering temperature plays a critical role in controlling the densification of TZP walls while preserving the elongated microporous structures generated by the PS-VP process.

**Figure 9:**
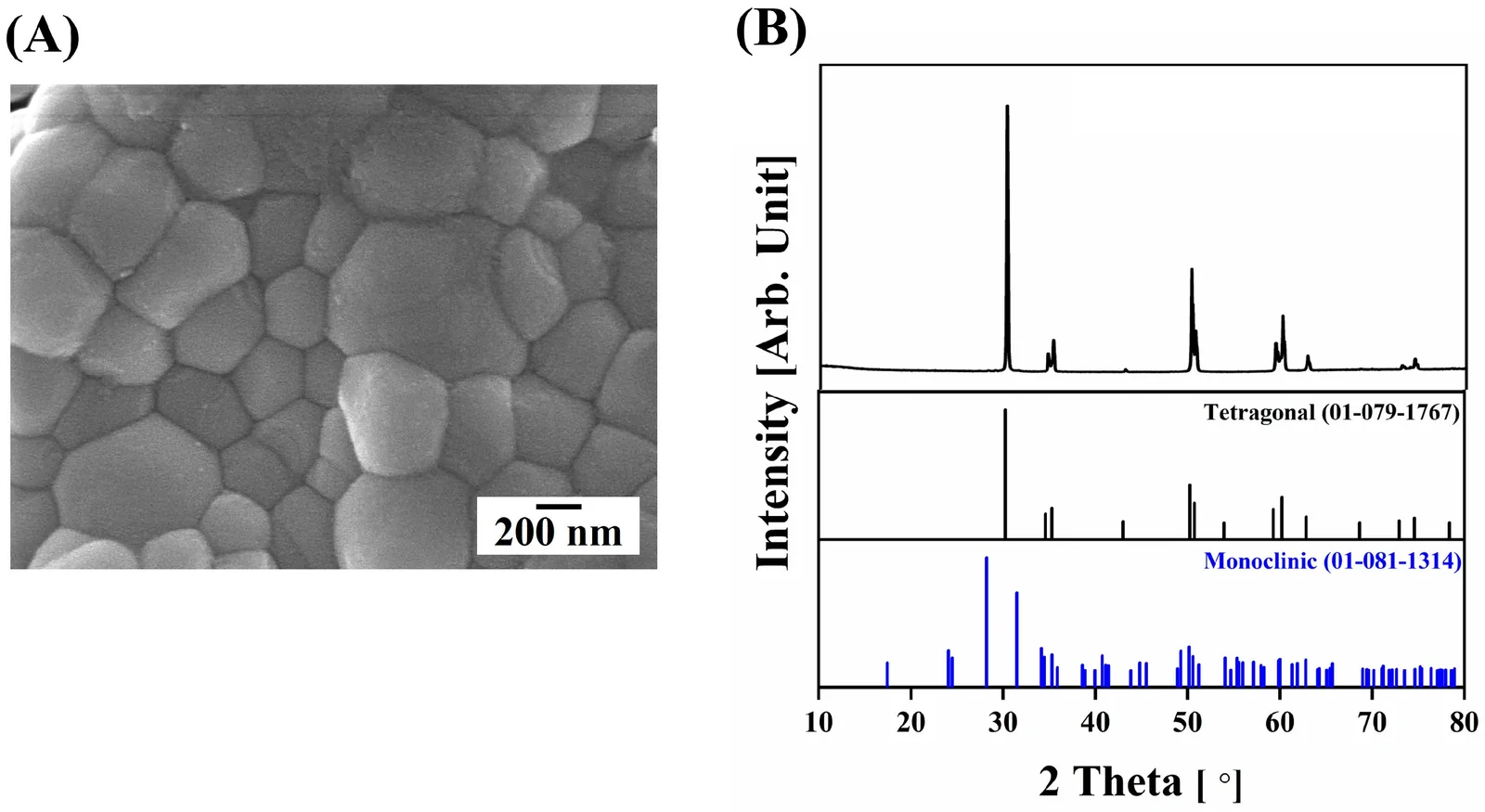
(**A**) FE-SEM image and (**B**) XRD pattern of TZP walls sintered at 1500 °C for 3 h.

The densification of the TZP walls sintered at 1500 °C is shown in Figure 9A. The TZP walls could be almost fully densified even using a ceramic loading as low as 7.83 vol% in the TZP suspension. Specifically, the TZP particles with HDDA monomer were effectively ejected by growing camphene dendrites and subsequently concentrated within the interdendritic spacing. This resulted in a high ceramic content of ~26.1 vol% in the HDDA/TZP walls, thereby allowing excellent densification. However, it should be noted that higher ceramic contents (TZP powder contents) in TZP suspensions are beneficial to densification, but reduce the camphene content and the resulting porosity. The TZP walls are composed of fine grains with an average size of 643 ± 87.5 nm.

The XRD pattern of the microporous TZP scaffold sintered at 1500 °C for 3 h is shown in Figure 9B. All strong peaks were indexed to the tetragonal phase. In addition, no peaks were observed at 2θ = 28.2° and 31.3°, which are the strong peaks of the monoclinic phase. The monoclinic phase fraction, calculated by Rietveld refinement, was as low as 0.8 wt%. This finding suggests that the TZP walls are mainly composed of the tetragonal-phase grains with a negligible fraction of monoclinic-phase grains, thereby offering excellent mechanical properties and resistance to low-temperature degradation.

**Figure 10:**
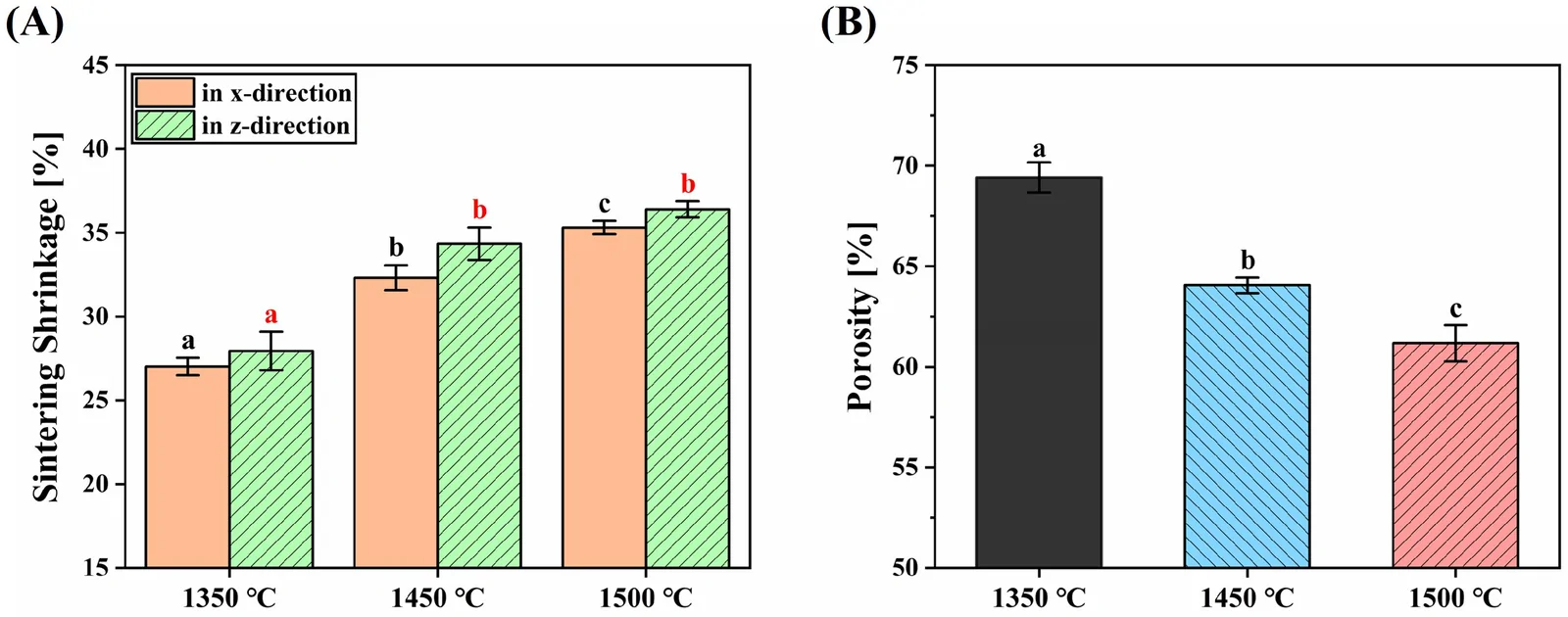
(**A**) Sintering shrinkage and (**B**) porosity of microporous TZP samples after sintering for 3 h at various temperatures (1350 °C, 1450 °C, and 1500 °C). Different subscript letters indicate statistically significant differences between groups according to Tukey’s post-hoc test (*p*-value < 0.05). For the shrinkage data in (**A**), black and red letters correspond to the x- and z-direction, respectively.

The sintering shrinkages of microporous TZP samples in the x- and z-directions are shown in Figure 10A. All samples exhibited slightly greater sintering shrinkage in the z-direction, parallel to the layer-by-layer PS-VP direction, compared with the x-direction, which is a typical feature of ceramic VP [45,50]. The shrinkage increased with increasing sintering temperature, which is consistent with the enhanced densification of TZP walls observed in the FE-SEM images (cf., Figure 8). In the x-direction, the shrinkage increased from 27.02 ± 0.52% to 32.32 ± 0.75% and 35.31 ± 0.40% as the sintering temperature increased from 1350 °C to 1450 °C and 1500 °C, respectively. Significant differences were observed among all sintering temperatures (one-way ANOVA, *p* = 6.11 × 10^−6^) according to Tukey’s post-hoc test. In the z-direction, the shrinkage also increased from 27.95 ± 1.15% at 1350 °C to 34.35 ± 0.97% at 1450 °C and 36.40 ± 0.48% at 1500 °C. A significant difference was observed between 1350 °C and 1450 °C (*p* = 7.17 × 10^−5^, Tukey’s post-hoc test) according to Tukey’s post-hoc test. However, it should be noted that no significant difference was observed between the samples sintered at 1450 °C and 1500 °C (*p* = 0.0754, Tukey’s post-hoc test). This finding suggests that most of the densification-induced shrinkage in this direction occurred between 1350 °C and 1450 °C.

The porosity, attributed to elongated micropores and residual small pores within the TZP walls, decreased with increasing sintering temperature, as shown in Figure 10B. The samples sintered at 1350 °C, 1450 °C, and 1500 °C exhibited porosities of 69.42 ± 0.75%, 64.05 ± 0.39%, and 61.17 ± 0.90%, respectively. Statistical analysis showed significant differences among all sintering temperatures (one-way ANOVA, *p* = 2.25 × 10^−5^), indicating that the reduction in porosity was highly dependent on the sintering temperature [48]. This result is also in good agreement with the FE-SEM observations (cf., Figure 8), where the number of residual pores within the TZP walls decreased and the wall structure became denser as the sintering temperature increased. Based on these observations, a sintering temperature of 1500 °C with a dwelling time of 3 h was employed to fabricate unidirectionally macrochanneled TZP scaffolds composed of microporous frameworks.

### 3.7. Macroporous and Microporous Structures of Unidirectionally Macrochanneled TZP Scaffolds with Elongated Microporous Frameworks

**Figure 11:**
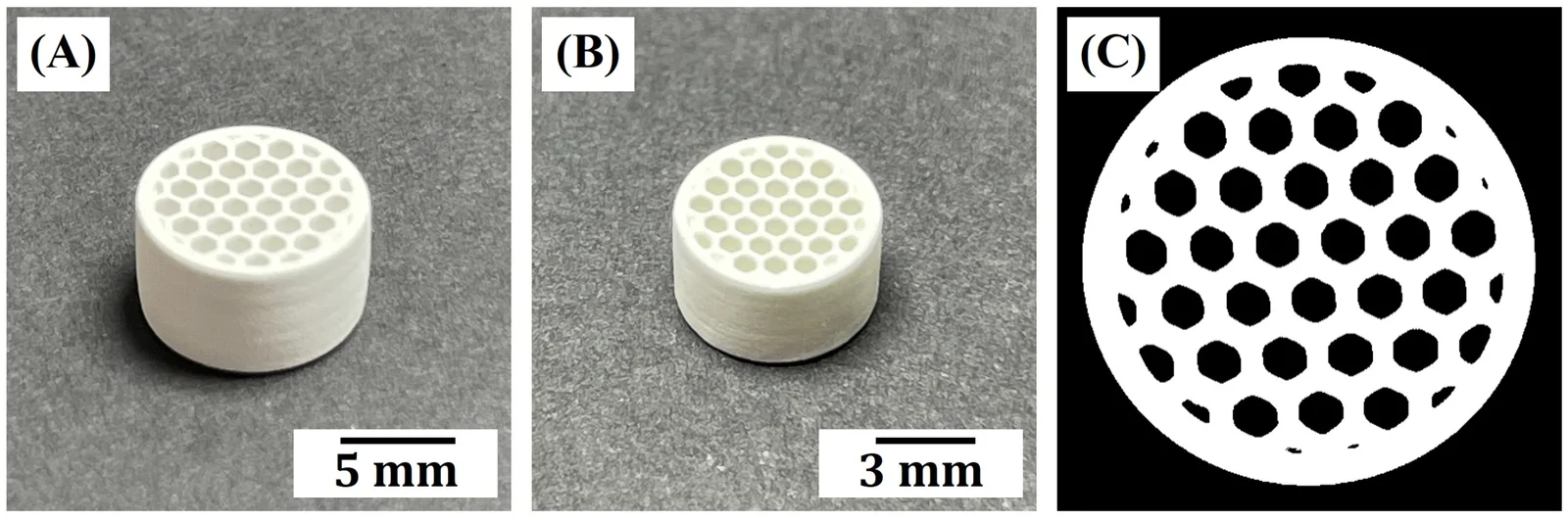
Optical images of the unidirectionally macrochanneled TZP scaffolds with elongated microporous frameworks (**A**) before and (**B**) after sintering at 1500 °C for 3 h, and (**C**) a representative digitally colored image obtained from a top-view OM image.

To demonstrate the utility of the PS-VP process in fabricating mechanically robust porous ceramic scaffolds for potential load-bearing bone scaffold applications, unidirectionally macrochanneled TZP scaffolds with elongated microporous frameworks were produced using the PS-VP process. As shown in Figure 11A, the resulting green body exhibited a well-defined cylindrical shape with unidirectional macrochannels arranged in a regular hexagonal pattern. No visible defects at the interfaces between the layers were observed owing to the optimum processing parameter for the PS-VP process, including a UV illumination time of 20 s for a layer thickness of 100 μm. After debinding and subsequent sintering at 1500 °C for 3 h, the sintered scaffold retained the original honeycomb-like macrochannel architecture without any noticeable defects, such as cracks at the interfaces between layers or within the layers (Figure 11B). To more clearly examine the construction of periodic hexagonal macrochannels, the top surfaces of the scaffolds were captured by optical microscopy and then digitally colored using ImageJ software for clearer visualization. Tightly controlled macrochannels were uniformly created throughout the scaffolds, as shown in Figure 11C.

Table 2 summarizes the overall porosity, macrochannel size, and framework width of the unidirectionally macrochanneled TZP scaffolds with elongated microporous frameworks after sintering at 1500 °C for 3 h. A high overall porosity of 74.65 ± 0.73% was obtained owing to the creation of numerous micropores within the TZP frameworks along with the macrochanneled structure. The macrochannel size and framework width were estimated based on the digitally thresholded images (cf., Figure 11C). During sintering at 1500 °C for 3 h, the scaffolds shrank considerably, resulting in a much smaller macrochannel size (0.99 ± 0.02 mm) than that of the CAD model (1.7 mm, cf. Figure 1B). Meanwhile, the framework width was 267 ± 24 μm, slightly larger than that of the CAD model (250 μm) mainly due to the line broadening effect (cf., Figure 6B). Note that the small deviations observed in the macrochannel size and framework width indicate the high dimensional accuracy of the PS-VP process. Consequently, the macrochannel size and framework width of sintered scaffolds can be reasonably tailored by adjusting the dimensions of initial CAD models to balance the overall porosity and mechanical properties if necessary.

**Figure 12:**
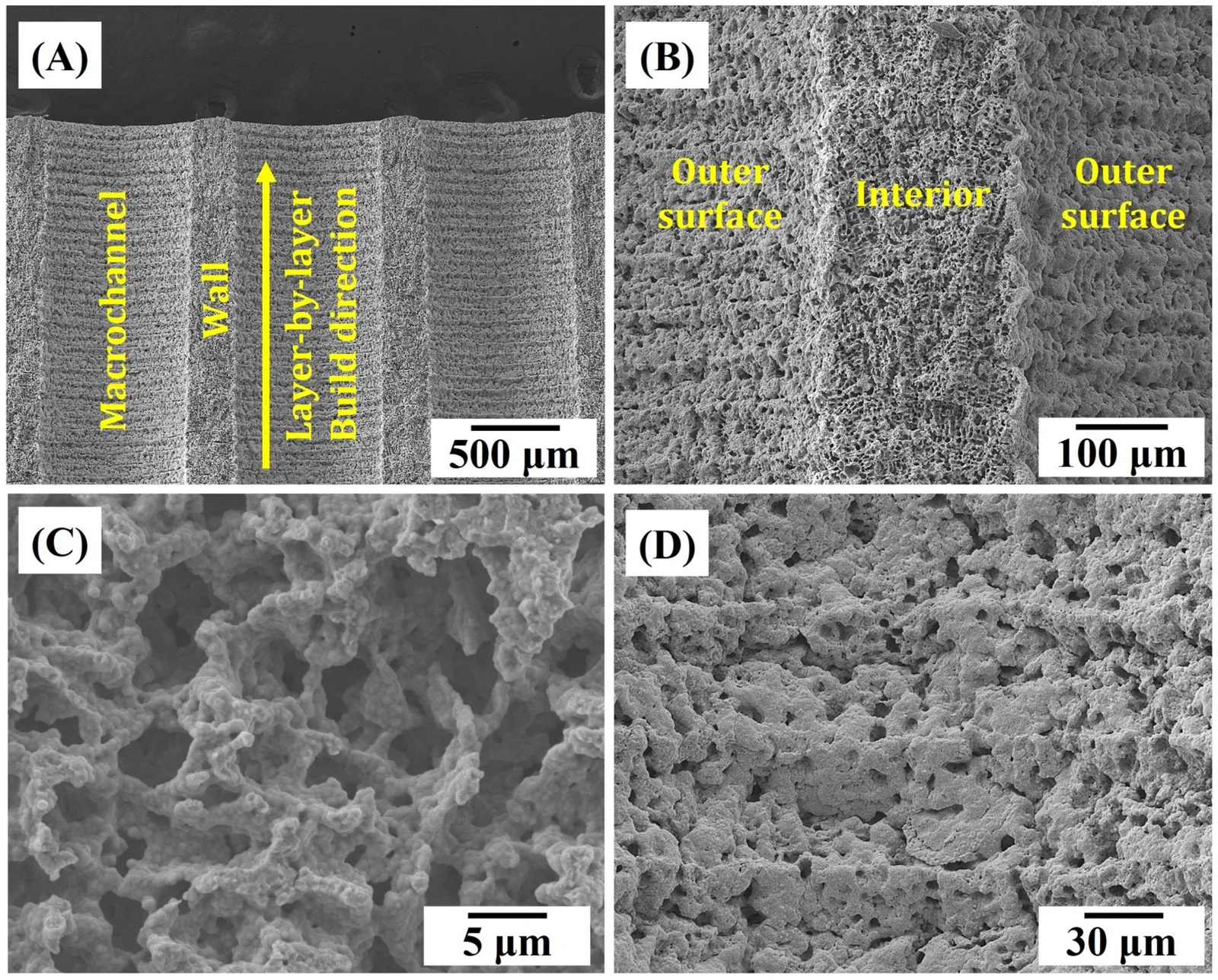
FE-SEM images of the fractured macrochanneled TZP scaffold showing (**A**) the microporous frameworks surrounding straight macrochannels, (**B**) the interior and outer surfaces of the framework, (**C**) the porous structure of the interior, and (**D**) the porous structure of the outer surface.

The macroporous and microporous structures of the unidirectionally macrochanneled TZP scaffolds with elongated microporous frameworks were examined by FE-SEM, as shown in Figure 12A–D. Straight macrochannels surrounded by microporous frameworks were uniformly formed throughout the scaffolds (Figure 12A). In addition, the PS-VP-processed layers were well bonded together with shallow grooves at the interfaces. Figure 12B shows the interior and outer surfaces exposed to the macrochannels. A number of elongated micropores with good pore interconnectivity were created within the TZP frameworks (Figure 12C), which were analogous to those obtained from the disks (cf., Figure 8E,F). However, the outer surfaces were less porous (Figure 12D), which was presumably due to the rapid crystallization of camphene dissolved in the TZP suspension. Nevertheless, the TZP walls enclosing the micropores throughout the interior and outer surfaces were highly densified owing to sintering at 1500 °C for 3 h.

### 3.8. Mechanical Properties of Unidirectionally Macrochanneled TZP Scaffolds with Elongated Microporous Frameworks

**Figure 13:**
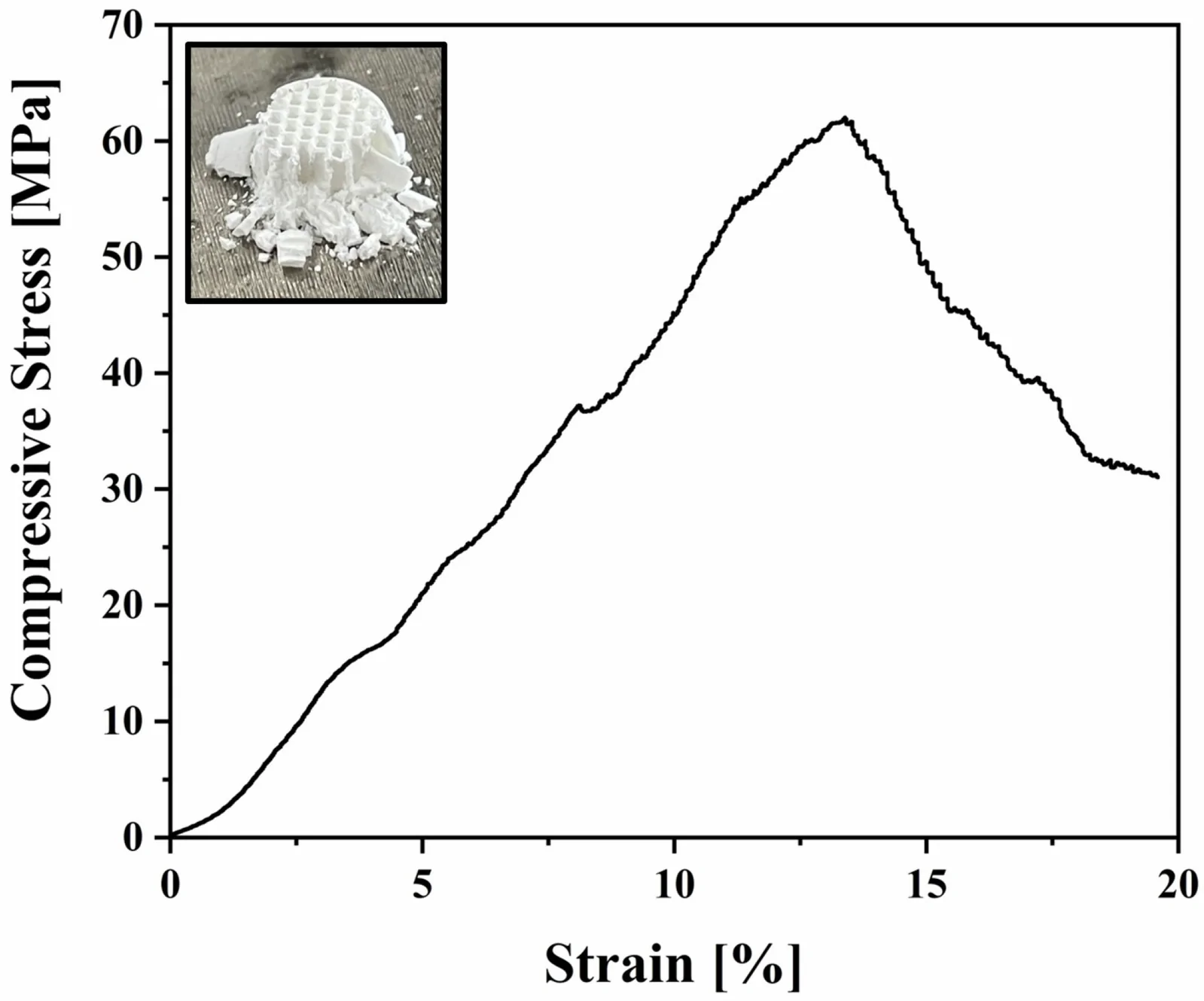
Representative compressive stress–strain curve of the unidirectionally macrochanneled TZP scaffold with elongated microporous frameworks, measured under compressive loading parallel to the macrochannel direction. The inset shows the fractured scaffold after the compressive test.

The mechanical properties of the unidirectionally macrochanneled TZP scaffolds with elongated microporous frameworks were characterized by compressive strength tests. As shown in Figure 13, the scaffold exhibited a fracture behavior in which the compressive stress increased almost linearly with increasing strain until reaching the maximum value. No pronounced serrations, characterized by repeated decreases and increases in stress, were observed before reaching the maximum point, although the scaffold was composed of highly microporous frameworks. This result indicates that the unidirectionally aligned TZP frameworks effectively withstood the applied compressive load when compressed parallel to the macrochannel direction [32,33,42]. After the maximum point, the stress decreased gradually. This behavior was attributed to the successive breakage and crushing of the microporous frameworks (the inset of Figure 13). More specifically, the remaining neighboring frameworks continued to support the applied load to a certain extent, preventing an immediate overall collapse [51,52,53]. In addition, the elongated micropores within the frameworks, oriented along the macrochannel direction, contributed to an excellent load-bearing capacity. More specifically, the mechanical properties of porous ceramics are highly dependent on pore geometry. When unidirectionally compressed along the direction of elongated pores with high aspect ratios, the preferentially oriented TZP walls can more effectively transfer applied loads to neighboring walls, thereby withstanding a higher overall load before fracture. These excellent load-transfer and load-bearing capabilities offer superior compressive strength compared to those of scaffolds with uniform, spherical pores, which are often observed in the unidirectional freeze casting of porous ceramics [54,55,56,57]. However, it should be noted that the anisotropic pore architecture results in lower compressive strength when compressed normal to the pore direction. Therefore, it is critical to create elongated pores along the unidirectionally aligned TZP frameworks to ensure superior mechanical properties.

Table 3 summarizes the compressive strength and compressive modulus, calculated from the maximum point and slope of the compressive stress–strain curves, respectively. Notably a high compressive strength (53.10 ± 8.19 MPa) and compressive modulus (364.38 ± 70.90 MPa) were obtained even at a high overall porosity (74.65 ± 0.73%). These high values were attributed to both the creation of the unidirectionally aligned TZP frameworks with elongated micropores and the achievement of highly densified TZP walls surrounding the micropores. This finding suggests that the unidirectionally macrochanneled TZP scaffolds with elongated microporous frameworks, fabricated using the PS-VP process, offer great potential as load-bearing bone scaffolds to replace bones, such as vertebrae and femurs, which experience considerable compressive loads during their physiological functions [17,18,34,35].

The present study has demonstrated that the PS-VP process can create unidirectionally aligned macrochannels surrounded by microporous TZP frameworks comprising elongated micropores in a tightly controlled manner. Consequently, the dual-scale macro-/micro-porous TZP scaffolds exhibit excellent compressive strength at a given porosity, when compressed along the macrochannel direction. However, when compressed normal to the macrochannel direction, a much lower compressive strength is inevitable. Although this study aims to provide a useful strategy for producing high-strength porous scaffolds, it is necessary to further optimize pore geometry and orientation, particularly considering complex loads, including not only compressive stress but also shear stress applied to load-bearing bones. Furthermore, to verify the practical bone scaffold applications of these dual-scale macro-/micro-porous TZP scaffolds, carefully designed in vivo animal experiments should be conducted with a special focus on bone regeneration along long, straight unidirectional macrochannels.

## 4. Conclusions

The PS-VP process successfully produced unidirectionally macrochanneled TZP scaffolds with elongated microporous frameworks, exhibiting excellent mechanical properties even at high porosities. A number of elongated micropores were created within the TZP frameworks, which were replicas of camphene crystals formed via the phase separation of the TZP suspensions. Employing a sintering process at 1500 °C for 3 h enabled the nearly full densification of TZP walls, while preserving the elongated pore geometry, resulting in a high microporosity (61.17 ± 0.90%). Consequently, the TZP scaffolds exhibited a high overall porosity of 74.65 ± 0.73%. The combination of unidirectionally aligned TZP frameworks surrounding the macrochannels and elongated micropores within the frameworks enabled the achievement of excellent mechanical properties: a compressive strength of 53.10 ± 8.19 MPa and a compressive modulus of 364.38 ± 70.90 MPa. These results indicate that the PS-VP process provides opportunities to significantly enhance mechanical properties at a given porosity, rendering the scaffolds a promising candidate for further investigation in load-bearing bone scaffold applications.

## Figures and Tables

**Table 1:** Compositions and constituent volume fractions of TZP suspension for PS-VP process.

Role	TZP Powder	Pore-Forming Agent	Photocurable Monomer	Dispersant	Photoinitiator
Constituent	3YS-E	Camphene	HDDA	DISPERBYK-180	TPO
Vol %	7.83	70.00	19.45	2.19	0.53

**Table 2:** The overall porosity, macrochannel size, and framework width of the unidirectionally macrochanneled TZP scaffolds with elongated microporous frameworks after sintering at 1500 °C for 3 h.

Overall Porosity [Vol %]	Macrochannel Size [mm]	Framework Width [μm]
74.65 ± 0.73	0.99 ± 0.02	267 ± 24

**Table 3:** Compressive strength and compressive modulus of unidirectionally macrochanneled TZP scaffolds with elongated microporous frameworks.

Compressive Strength [MPa]	Compressive Modulus [MPa]
53.10 ± 8.19	364.38 ± 70.90

## Data Availability

The original contributions presented in this study are included in the article. Further inquiries can be directed to the corresponding author.
